# A Rare Case of Pneumobilia and Pancreatitis Following Difficult IRIS‐Guided Nasoenteric Tube Placement With Additional Air Insufflation: A Case Report and Literature Review

**DOI:** 10.1155/crgm/1251527

**Published:** 2026-06-07

**Authors:** Alaa Alresheq, Mustafa A. Shahrori, Mohammad Almeqdadi

**Affiliations:** ^1^ Department of Internal Medicine, Al-Quds University, East Jerusalem, P600, State of Palestine, alquds.edu; ^2^ Department of Transplant Hepatology, Tufts Medical Center, Boston, Massachusetts, 02111, USA, tuftsmedicalcenter.org; ^3^ Faculty of Medicine, An-Najah National University, Nablus, State of Palestine, najah.edu

**Keywords:** insufflation, nasogastric tube, pancreatitis, pneumobilia

## Abstract

This article presents a rare case of acute pancreatitis and pneumobilia identified after difficult Kangaroo feeding tube with Integrated Real‐time Imaging System (IRIS) Technology insertion in a 40‐year‐old female with multiple comorbidities. The patient’s profound cardiogenic shock, recently initiated captopril, and imaging evidence of descending and sigmoid colitis suggested a likely multifactorial process that may also have included ischemic pancreatic and bowel injury. Although a temporal association with additional air insufflation was noted, the exact causal relationship remains uncertain, with a procedure‐related contribution considered more plausible for pneumobilia than for pancreatitis. To our knowledge, no similar published case was identified. The article highlights the possible etiologies of these complications after NG tube insertion and emphasizes the need for careful insufflation practices and thoughtful deployment of advanced NG tube technologies in critically ill patients.

## 1. Introduction

Pneumobilia, the presence of gas within the biliary tree, is typically iatrogenic and most commonly encountered after biliary instrumentation, particularly after endoscopic retrograde cholangiopancreatography (ERCP) and biliary sphincterotomy. It has been associated with emphysematous cholecystitis, ascending cholangitis, spontaneous biliary‐enteric fistula, biliary bronchopleural fistula, and visceral blunt trauma. Importantly, pneumobilia is a radiologic finding rather than a diagnosis in itself, and its clinical significance depends on the underlying cause and overall clinical context [1].

Nasogastric and nasoenteric tubes are commonly used in the management of critically ill patients for nutritional support and gastric decompression. Tube placement is generally considered safe; however, recognized complications include discomfort, sinusitis, epistaxis, malposition, aspiration, perforation, and airway injury. These risks underscore the importance of careful technique, appropriate confirmation of position, and close clinical vigilance, particularly in medically complex patients [2, 3].

The Kangaroo feeding tube with Integrated Real‐time Imaging System (IRIS) Technology was developed to improve placement accuracy by allowing real‐time visualization of anatomic landmarks during insertion. Clinical studies of IRIS‐guided placement have shown that the system can assist with recognition of the upper aerodigestive tract, esophagus, stomach, and small bowel and may facilitate earlier identification of respiratory misplacement compared with conventional blind advancement [4–7].

After review of the literature, pneumobilia and acute pancreatitis do not appear to be typical complications of nasogastric or nasoenteric tube insertion. We report an unusual case in which these findings developed after difficult placement of a Kangaroo feeding tube with IRIS Technology and documented additional air insufflation during postpyloric positioning. This case highlights a temporal association between a technically challenging tube‐placement procedure and subsequent abdominal complications in a critically ill patient [2, 4–7].

## 2. Case Presentation

A 40‐year‐old woman with a past medical history of poorly controlled Type 1 diabetes mellitus and polysubstance abuse, including heroin, opioids, and cocaine, with no history of heavy alcohol use, initially presented with inability to move her left upper extremity, retrosternal chest pain associated with diaphoresis, myalgia, and low‐grade fever. She had reportedly been feeling generally unwell for the preceding 5 days and had a positive home COVID‐19 test.

At the referring hospital, her troponin level was reported to be greater than 20,000. Cardiac catheterization was initially planned; however, because she was tachypneic and hypotensive, pulmonary embolism was suspected. Computed tomography pulmonary angiography reportedly demonstrated a subsegmental pulmonary embolism, and she was started on intravenous heparin. She also received levofloxacin 750 mg intravenously once daily and piperacillin–tazobactam 3g intravenously every 6 h because of concern for possible infection in the setting of hemodynamic instability. Computed tomography of the head showed no acute intracranial findings.

She was subsequently transferred to another center, where electrocardiography demonstrated right bundle branch block, complete heart block, a heart rate of 40–60 beats/min, and ST‐segment elevation in V2. An anterior ST‐elevation myocardial infarction was suspected. Coronary angiography revealed complete thrombotic occlusion of the left anterior descending artery, and she underwent percutaneous coronary intervention. Her subsequent course was complicated by severe cardiogenic shock requiring mechanical circulatory support and inotropic therapy.

Additional laboratory abnormalities included blood glucose greater than 600 mg/dL, requiring insulin infusion, aspartate aminotransferase greater than 31,000 U/L, alanine aminotransferase rising to 4000 U/L from approximately 1000 U/L, INR of 1.5, and troponin downtrending from approximately 25,000 to 9000. During this course, she also developed a right femoral artery thrombus requiring thrombectomy. She was then admitted to our intensive care unit (ICU), sedated, and mechanically ventilated, for continued management of cardiogenic shock.

During her ICU course, captopril was initiated at 6.25 mg orally three times daily. A Kangaroo feeding tube with IRIS was inserted for enteral feeding. According to the procedure notes, placement of the Dobhoff tube was challenging and additional air was insufflated into the stomach during the procedure. The procedure duration was approximately 5–7 min. Air was insufflated intragastrically to distend the stomach, and the gastric structures were visualized after 5‐6 min of repeated insufflation. No forceful advancement maneuvers or external abdominal compression were used. The tube was documented as being proximal to the ampulla. She had no history of prior ERCP, sphincterotomy, or upper endoscopy.

**FIGURE 1:**
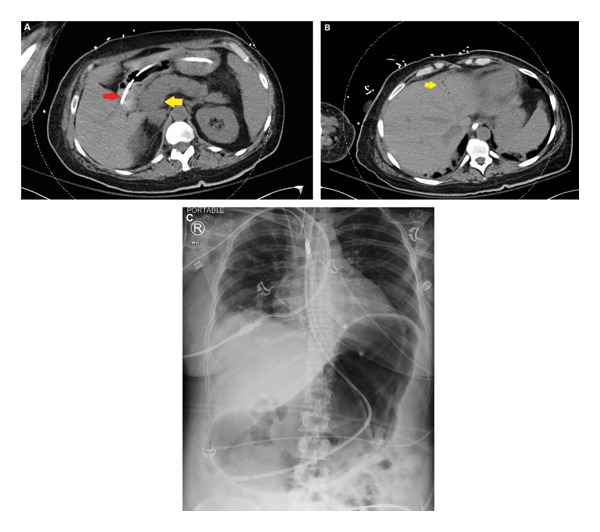
(A) CT scan of the abdomen with yellow arrowhead showing pancreatic head with edema and evidence of acute pancreatitis and red arrowhead showing the NG tube tip in the duodenum. (B) CT scan of the abdomen with yellow arrowhead showing pneumobilia. (C) Abdominal X‐ray showing distended stomach after NG tube insertion.

Subsequently, the patient developed abdominal pain with elevated serum lipase. Triglyceride, calcium, and IgG levels were within normal limits. Computed tomography and ultrasonography demonstrated acute uncomplicated pancreatitis, descending and sigmoid colitis, and intrahepatic biliary gas consistent with pneumobilia. Major clinical events are summarized in Table 1. Figure 1A shows pancreatic head edema with findings consistent with acute pancreatitis, with the tube tip seen in the duodenum. Figure 1B demonstrates intrahepatic biliary gas consistent with pneumobilia. Figure 1C shows a distended stomach on abdominal X‐ray after the tube insertion.

**TABLE 1:** Summary timeline of major clinical events.

Stage	Event
Initial presentation	Left upper extremity weakness, chest pain, diaphoresis, myalgia, low‐grade fever, positive home COVID‐19 test
Early evaluation	Troponin > 20,000; suspected pulmonary embolism; heparin started
Cardiac diagnosis	Anterior STEMI diagnosed after transfer
Intervention	Coronary angiography and PCI performed
Subsequent course	Cardiogenic shock requiring mechanical and inotropic support
Additional complication	Right femoral artery thrombus requiring thrombectomy
ICU course	Captopril initiated
Feeding‐tube procedure	Difficult IRIS‐guided Dobhoff placement with repeated gastric air insufflation
After procedure	Abdominal pain and elevated serum lipase developed
Imaging findings	Acute pancreatitis, pneumobilia, and descending/sigmoid colitis

Abbreviations: ICU, intensive care unit; IRIS, integrated real‐time imaging system; PCI, percutaneous coronary intervention; STEMI, ST‐elevation myocardial infarction.

In view of the temporal relationship between the difficult tube placement with documented additional air insufflation and the subsequent imaging findings, a procedure‐related contribution to the pneumobilia was considered. However, the etiology of pancreatitis remained uncertain, and drug‐induced pancreatitis related to recently initiated captopril was also considered.

## 3. Discussion

This case describes pneumobilia and acute pancreatitis identified after difficult placement of a Kangaroo feeding tube with IRIS Technology in a critically ill patient with multiple comorbidities. The close temporal relationship between tube placement and the subsequent abdominal findings raises concern for a procedure‐related contribution, particularly with respect to pneumobilia; however, causality cannot be definitively established.

The Kangaroo feeding tube with IRIS Technology is a direct‐visualization system rather than a conventional blind bedside tube. It was designed to improve tube advancement by allowing the operator to visualize anatomic structures during insertion, thereby helping identify the airway, esophagus, stomach, and, in some cases, the small bowel. Published experience with IRIS‐guided placement supports its role in improving recognition of anatomy and reducing unrecognized respiratory misplacement, but it does not eliminate procedural risk, especially when advancement is technically difficult or the patient is medically unstable [4–7].

In the present case, the important procedural detail is not simply that a feeding tube was inserted but that Dobhoff placement was documented as challenging and that additional air was insufflated during an attempt at postpyloric positioning. This distinction is important because it clarifies that the event occurred during technically difficult postpyloric placement rather than routine blind bedside insertion. The concern is, therefore, not that IRIS technology itself is unsafe but that unusual complications may still occur despite advanced placement technology when insertion is difficult and the patient is clinically vulnerable [4–7].

More detailed procedural mechanics would have been helpful in assessing causality. The available documentation indicates that the procedure duration was approximately 5–7 min and that repeated intragastric air insufflation was used to distend the stomach and improve visualization, with gastric structures visualized after 5‐6 min of repeated insufflation. No forceful advancement maneuvers or external abdominal compression were documented. Although these details do not establish causality, they help frame the event as a technically challenging procedure involving repeated insufflation in a fragile patient.

Pneumobilia should be discussed separately from pancreatitis. Pneumobilia is not intrinsically dangerous in itself; rather, it is an imaging finding whose significance depends on the underlying etiology. In this patient, there was no history of ERCP, sphincterotomy, or upper endoscopic instrumentation, and imaging did not demonstrate gallstones or common bile duct dilatation. In that setting, the temporal link between difficult tube placement, documented additional air insufflation, and subsequent intrahepatic biliary gas supports the consideration of a procedure‐related contribution to the pneumobilia. A possible explanation is that increased local intragastric or duodenal pressure during difficult advancement and insufflation may have facilitated retrograde passage of air through the periampullary region into the biliary tree. This mechanism remains hypothetical and cannot be confirmed from a single case [1].

If pneumobilia is identified after difficult feeding‐tube placement, clinicians should first exclude more serious etiologies such as bowel ischemia, cholangitis, perforation, or biliary‐enteric fistula through clinical assessment and appropriate imaging. In clinically stable patients without evidence of biliary obstruction or sepsis, conservative management and close monitoring may be appropriate. Rare cases of aerobilia after enteral tube procedures have also been described in the literature, suggesting that this finding may occasionally occur in the setting of difficult instrumentation or insufflation‐related pressure changes [8].

On review of the available images, the gas pattern was most consistent with pneumobilia. Gas within the main pancreatic duct was not clearly identified, so pneumopancreas could not be confidently diagnosed. In addition, in a patient with profound shock and bowel injury, hepatic portal venous gas should also be considered in the differential diagnosis of intrahepatic gas. Although the distribution in this case was interpreted as pneumobilia, the broader differential remains clinically relevant [9].

The association with acute pancreatitis is more difficult to define. Although the patient developed abdominal pain and elevated lipase after the difficult tube‐placement procedure, and imaging was consistent with acute uncomplicated pancreatitis, the etiology remained uncertain. More common evaluated causes such as hypertriglyceridemia, hypercalcemia, autoimmune pancreatitis, gallstones, and biliary dilatation were reportedly not supported by the available data. Nevertheless, the absence of these findings does not prove that tube placement caused pancreatitis. The patient was critically ill, had recently undergone extensive cardiovascular intervention, and had been started on captopril shortly before the onset of abdominal findings. Captopril‐associated acute pancreatitis has been reported in the literature, and systematic review data suggest that drug‐induced pancreatitis is a recognized but variably supported entity. Accordingly, the pancreatitis in this case is best regarded as temporally associated with the procedure but of uncertain and likely multifactorial etiology [10–12].

Profound cardiogenic shock may also have contributed substantially to the abdominal findings. The pancreas is vulnerable to hypoperfusion, and ischemic acute pancreatitis has been described after severe hypotension and vascular compromise. Likewise, bowel ischemia or ischemic enteropathy may occur in the setting of shock and vasopressor exposure and can produce mural injury, altered bowel compliance, and portal venous gas. In the present case, the combination of severe shock, marked transaminase elevation consistent with shock liver, and imaging evidence of descending and sigmoid colitis makes an ischemic component biologically plausible.

The colitis finding, therefore, deserves explicit comment. Because the selected figures were intended to illustrate pancreatitis, pneumobilia, and gastric distention, they do not adequately depict the descending and sigmoid colitis described on computed tomography. Nevertheless, in the clinical context of cardiogenic shock and vasopressor requirement, this finding may reflect ischemic colitis or splanchnic hypoperfusion rather than an unrelated incidental abnormality. Its presence supports a broader multifactorial interpretation of the abdominal complications and may also help explain reduced bowel compliance during insufflation.

A further mechanistic possibility is a synergy between ischemic bowel dysfunction, gas insufflation, and local mechanical irritation. In severe shock, bowel atony and loss of normal sphincter tone may limit distal gas transit and permit a disproportionate rise in intraduodenal pressure even with standard insufflation. Under those conditions, retrograde reflux of air through the sphincter of Oddi could contribute to pneumobilia, while duodenal content reflux or local periampullary irritation might contribute to pancreatitis. Figure 1A also shows the tube tip in the duodenum, and migrated enteral tubes have been reported to cause pancreatitis through direct contact with or extrinsic compression near the ampulla of Vater. These mechanisms remain speculative in this patient but are anatomically plausible and support a multifactorial model rather than a single‐cause explanation [13–16].

The broader issue of tube‐placement safety also deserves emphasis. The BAPEN National Patient Safety Alerting Committee Position Paper on Nasogastric Tube Safety emphasizes recommended confirmation methods such as aspirate pH and/or X‐ray, and related BAPEN guidance states that the “whoosh test” (air auscultation) should not be used. Although the present case involved IRIS‐guided placement rather than routine blind confirmation, the same principle applies: tube placement should not be viewed as a trivial bedside intervention, and careful technique, clear documentation, and appropriate confirmation remain essential [3, 17].

This case also has practical implications for the treatment strategy in critically ill patients. When postpyloric feeding‐tube placement is technically difficult or the patient is profoundly unstable, clinicians may need to consider alternative approaches such as delaying further advancement, using gastric feeding temporarily when appropriate, or obtaining fluoroscopic or endoscopic assistance if the anticipated benefit outweighs the procedural risk. The choice should be individualized according to the hemodynamic status, aspiration risk, nutritional goals, and operator experience.

A historical report of fatal gastric rupture after overinflation through a stomach tube suggests that excessive insufflation can, in rare circumstances, be harmful. That literature should be interpreted cautiously, but it supports the broader point that insufflation is not always physiologically benign, particularly in vulnerable patients. In the present case, it is, therefore, reasonable to consider that additional air insufflation may have contributed to the development of pneumobilia, while any relationship to pancreatitis remains less certain [18].

## 4. Conclusion

This case highlights pneumobilia and acute pancreatitis as rare abdominal findings identified after difficult IRIS‐guided nasoenteric tube placement with additional air insufflation in a critically ill patient. A procedure‐related contribution to pneumobilia is plausible, whereas the pancreatitis was likely multifactorial and may have reflected the combined effects of difficult manipulation, duodenal tube position, recently initiated captopril, and severe cardiogenic shock with possible ischemic bowel injury. Careful insufflation technique, detailed procedural documentation, and individualized feeding‐tube placement strategies are warranted in similarly vulnerable patients.

## Funding

No funding was received for this manuscript.

## Consent

No written consent has been obtained from the patient as there is no patient identifiable data included in this case report.

## Conflicts of Interest

The authors declare no conflicts of interest.

## Data Availability

The data that support the findings of this study are available on request from the corresponding author. The data are not publicly available due to privacy or ethical restrictions.
